# Risk perception, motives and behaviours in university students

**DOI:** 10.1080/02673843.2014.919599

**Published:** 2014-06-11

**Authors:** P. Salameh, J. Salamé, M. Waked, B. Barbour, N. Zeidan, I. Baldi

**Affiliations:** ^a^ Clinical and Epidemiological Research Laboratory, Faculty of Pharmacy, Lebanese University, Hadath, Lebanon; ^b^ Charité, Universitätsmedizin University Hospital, Berlin, Germany; ^c^ Pulmonology Department, Faculty of Medicine, Saint Georges Hospital, Balamand University, Beirut, Lebanon; ^d^ Faculty of Public Health, Lebanese University, Fanar, Lebanon; ^e^ Laboratoire Santé Travail Environnement, Université Bordeaux Segalen, Bordeaux, France

**Keywords:** risk perception, risk benefit, motives, risk behaviour, university student

## Abstract

Risky behaviours among young people are relatively frequent, with several motives and attitudes lying behind. Our objective was to evaluate the role of risk perception, attractiveness and motives for risk behaviour taking among university students in Lebanon. A cross-sectional study was carried out using a proportionate cluster sample of Lebanese students in public and private universities. Items of risk intake and perception scale, attractiveness of risky behaviours, and motives for risky behaviours were assessed, in addition to cigarette and waterpipe smoking and dependence, alcohol problematic consumption and mental distress scale. After verifying the validity of scales and reliability in the university students' population, we found that risk perception was associated with lower risk intake, while risk attractiveness was a driver for it. Moreover, motives differed in their driving of risky behaviour, a particular point was that women indicated more goal achievement objectives, the latter concept was associated with lower risk taking. University students in Lebanon, women in particular, demonstrated wiser behaviour and may benefit from heath education programme to increase their awareness about risky behaviours. Identifying other personal, environmental, social and psychological predictors may also be important to improve effectiveness of these programmes.

## Introduction

Risky behaviours among young people are relatively frequent, and risk taking has been defined from several perspectives: from the decision-making perspective, Irwin defines it as ‘a volitional behavior whose outcome is uncertain and probably the reason of negative consequences’ (Irwin, 1990; Irwin & Millstein, 1991). Moore and Gullone (1996) defined risk taking behaviour as ‘behavior which involves potential negative consequences but is balanced in some way by perceived positive consequences’. Moreover, the reasons behind their risk taking have been explained by different theories and models (Igra & Irwin, 1996): the cognitive theory of risk taking is among the most popular and has investigated the development of decision-making capacities that potentially underlie risk-taking development, including sensitivity to risk, probability estimation and perceptions of vulnerability (Boyer, 2006). According to some researchers, adolescents often make rational decisions based on an appreciation of risk, and misjudgements are more likely to be the result of inexperience than of irrational decision-making, undeveloped cognitive abilities or a perception of personal invulnerability (Rodham, Brewer, Mistral, & Stallard, 2006).

Yet, risk taking has been presented as a precursor of problematic behaviour (Jessor & Jessor, 1977), leading to self destruction, psychological, social and health compromising situations (Ingersoll & Orr, 1989). However, risk taking has also been shown to be an important part of development into adulthood, particularly if it is goal directed (Jessor, 1991); risk taking is not merely for sensation seeking, but it sometimes has aims on a personal level (Shapiro, Siegel, Scovill, & Hays, 1998) or relational level (Engels & ter Bogt, 2001). A modest degree of risk-taking in adolescence seems to be normative and associated with some positive psychological characteristics (Shedler & Block, 1990).

To our knowledge, the majority of undertaken works on youth risk perception, motives and behaviours took place in developed countries; very few were published in the Middle Eastern Arab countries, Lebanon in particular. Because university students in Lebanon take risks frequently (Karam, Ghandour, Maalouf, Yamout, & Salamoun, 2010; Salameh et al., 2012a), and as risk taking is a culturally sensitive issue (Kloep, Guney, Cok, & Simsek, 2009), our objective was to evaluate the role of risk perception, attractiveness and motives for risk taking behaviour among university students in Lebanon.

## Methods

### Population and sampling

A cross-sectional study was carried out using a proportionate cluster sample of Lebanese students in public and private universities. A list of universities in Lebanon, provided by the Center for Pedagogic Researches, was used to adjust the sample size (Center for Educational Research and Development, 2010). A sample size of at least 3000 individuals was targeted to allow for adequate power for bivariate and multivariate analysis to be carried out.

Although ethical approval was granted by all Internal Review Boards of respective institutions where the study was conducted, most universities' administrative offices in Lebanon that we approached did not allow drawing a random sample of their enrolled students to participate in the study: they did not provide us with the lists of students and permission was not granted to enter classrooms and search for students nominatively. Thus, our research group had to work with a non-random sample of students outside their classes. Students were approached on campus during break times between courses by a field worker.

The latter explained the study objectives to the student, and after obtaining oral consent, the student was handed the anonymous and self-administered questionnaire. On average, the questionnaire was completed by participants within approximately 20 minutes. At the end of the process, the completed questionnaires were placed in closed boxes and sent for data entry. During the data collection process, the anonymity of the students was guaranteed. Out of 4900 distributed questionnaires, 3384 (69.1%) were returned to the field worker. Further methodological specifics are presented in more detail elsewhere (Salameh et al., 2012a; Salameh, Khayat, & Waked, 2012b; Salameh, Khayat, Waked, & Dramaix 2012c).

### Questionnaires

The questionnaire used in this study was composed of several parts, including the socio-demographic part, and a detailed active and passive smoking history. For risk perception assessment, risk involvement and perception scale (RIPS) score items were used (Mantzouranis & Zimmermann, 2010; Shapiro et al., 1998; Siegel et al., 1994); additional items that were deemed adapted to the local culture by the research group were also included: for every item, a Likert scale question was asked about how ‘dangerous’ the behaviour was considered by the university student, ranging from 0 (not at all) to 4 (very much). Moreover, questions about perceived benefit from risky behaviours and how ‘attractive’ the same items were found by university students were asked. A scale of ‘attractiveness of risky behaviours’ (ARB) was thus constructed, using Likert scale evaluation ranging from 0 (not at all) to 4 (very much). Furthermore, ‘motives for risk taking’ (MRT) were evaluated using items from a cross-cultural scale (Kloep et al., 2009).

Socio-economic status was evaluated using the ratio of mean income per household over the number of persons in the family. Mental distress was measured using the Beirut Distress Scale-22 (BDS22) (Salameh & Barbour, 2011). Current cigarette and waterpipe consumption were evaluated. For cigarette dependence, we assessed the Young Adults Cigarette Dependence (YACD) scale (Salameh et al., 2013) and for waterpipe dependence, the Lebanese Waterpipe Dependence Scale-11 (LWDS-11) (Salameh, Waked, & Aoun, 2008), both of which were developed by our team for the Lebanese population. The YACD was developed for university students; it is composed of 16 items, loading over six factors: nicotine dependence, craving intensity, positive reinforcement and negative reinforcement (Salameh et al., 2013).

### Statistical analysis

Data were entered and analysed using SPSS, version 18.0. Spearman correlation coefficients were calculated for scales and subscales correlations.

Factor analyses using the principal component analysis method were performed to allow for items loading over subscales of respective scales: ‘RIPS’, ‘ARB’ and ‘MRT’. For each factor analysis, Kaiser–Meyer–Oklin (KMO) measure of sampling adequacy was calculated, along with Bartlett's test of sphericity. Items with communality < 0.3 or loading on factors < 0.4 were removed from the scales. Pattern matrix after Promax rotation was reported because factors were correlated. Reliability of scales and subscales was also evaluated using Cronbach's α measurement.

Afterwards, the scales were face validated using several risky behaviours as dependent variables (current smoking cigarettes, current cigarette dependence, current waterpipe smoking, current waterpipe dependence and problematic use of alcohol), the validated scales (RIPS, ARB and MRT) as major independent variables and studying in a private university, age, sex, field of studies, region, mental distress (BDS22) and socio-economic status as covariates. For these purposes, multivariate stepwise descending likelihood ratio logistic regressions (in case of dichotomous dependent variables) and stepwise multiple regressions (in case of continuous dependent variables) were used. Application conditions were verified before the final models were accepted.

## Results

### Risk involvement and perception scale

For the RIPS, the KMO measure of sampling adequacy was 0.922, Bartlett's test of sphericity was *p* < 0.001 and 54.43% of variance were explained by the items. Out of 38 risky behaviours, 31 items were considered dangerous by university students and loaded on seven scales, explaining 54% of the total variance (Table 1). The first factor included most dangerous defying authorities or law-breaking behaviours, such as stealing, damaging properties or gambling for money. The second factor included drinking and driving behaviours, the third factor encompassed items about calculated risk, such as defending an unpopular idea and volunteering to speak in front of a group. The fourth factor was about minor academic misconduct such as skipping courses and cheating during an exam. The fifth factor was about exaggeration behaviours such as binge eating and having sex with more than one person, the sixth factor about speeding in a car and driving without a seatbelt. Finally, the seventh factor was about smoking cigarettes and waterpipes. Reliability measure for the total scale was 0.894, while those of subscale ranged between 0.921 for factor 1 (most dangerous behaviours) and 0.440 for the fifth factor (exaggeration behaviours) (Table 1).

**Table 1:** Risk intake and danger perception (RIPS) items (*n* = 31 items).

Items considered ‘dangerous’	Factor 1	Factor 2	Factor 3	Factor 4	Factor 5	Factor 6	Factor 7	Cronbach's α
Nicking something from a shop	0.865							0.921
Stealing something in a restaurant	0.852							
Damaging something such as a public phone	0.827							
Cheating in a school project	0.713							
Gambling for money	0.688							
Defying authorities	0.640							
Carrying weapons	0.605							
Getting involved in a fight	0.575							
Accepting ride from a stranger	0.507							
Hitchhiking	0.491							
Deliberately annoying somebody	0.462							
Consuming cocaine	0.427							
Taking chances in street traffic	0.409							
Getting drunk		0.751						0.739
Driving after alcohol drinking		0.745						
Being in a car with a drunk driver		0.634						
Drinking alcohol		0.606						
Defending an unpopular idea			0.841					0.700
Volunteering to speak in front of a group			0.798					
Organising an activity for friends			0.730					
Skipping courses				0.725				0.592
Cheating on an exam				0.608				
Not studying on an exam				0.500				
Taking a sunbath				0.481				
Binge eating					0.731			0.440
Having sex with more than one person					0.651			
Driving a car						0.821		0.521
Speeding in a car						0.622		
Driving without a seatbelt						0.559		
Smoking waterpipe							0.851	0.760
Smoking cigarettes							0.796	
Correlation with total scale	0.896	0.538	0.263	0.695	0.517	0.444	0.487	*p* < 0.001 for all

### Attractiveness of risky behaviours

For the ARB scale, the KMO measure of sampling adequacy was 0.932, Bartlett's test of sphericity was *p* < 0.001 and 57.74% of variance were explained by the items. Out of 38 items, 34 items were considered attractive by university students and loaded over eight factors, explaining 57.74% of the total variance of ARB (Table 2): the first factor involved ‘getting away’ behaviours such as stealing, damaging a public apparatus or cheating in a school work; the second factor was about ‘facing danger’ behaviours, such as contact sports, carrying weapons, fight involvements and so on. The third factor mainly involved licit and illicit substances consumption (alcohol, drugs, medications, …). The fourth factor included calculated responsible behaviours, the fifth factor was about sexual activity, the sixth factor was about relaxing behaviours (smoking and sunbathing), the seventh was about minor academic misconduct and the final eighth factor was about driving and speeding. Reliability α was 0.91 for the full scale, ranging from 0.877 for getting away behaviours to 0.479 for minor academic misconduct (Table 2).

**Table 2:** ARB items (*n* = 34 items).

Items considered ‘attractive’	Factor 1	Factor 2	Factor 3	Factor 4	Factor 5	Factor 6	Factor 7	Factor 8	Cronbach's α
Nicking something from a shop	0.857								0.877
Stealing in a restaurant	0.801								
Damaging something such as a public phone	0.778								
Hitchhiking	0.656								
Accepting ride from a stranger	0.651								
Cheating in a school work	0.447								
Contact sport participation		0.716							0.840
Carrying a weapon		0.701							
Getting involved in a fight		0.681							
Defying authorities		0.674							
Riding a motorcycle		0.635							
Taking chances in street traffic		0.630							
Deliberately annoying somebody		0.618							
Driving after alcohol drinking			0.720						0.825
Being in a car with a drunk driver			0.639						
Getting drunk			0.589						
Misusing prescription drugs			0.571						
Consuming cocaine			0.554						
Binge eating			0.540						
Crash dieting or taking diet pills			0.528						
Volunteering to speak in front of a group				0.812					0.745
Organising an activity for friends				0.750					
Defending an unpopular idea				0.741					
Having sex with a condom					0.871				0.635
Having sex without a condom					0.776				
Having sex with more than one person					0.523				
Smoking waterpipe						0.698			0.585
Smoking cigarettes						0.678			
Taking a sunbath						0.458			
Skipping courses							0.854		0.479
Cheating on exams							0.510		
Driving a car								0.769	0.550
Speeding in a car								0.551	
Driving without a seatbelt								0.434	
Correlation with total scale	0.786	0.834	0.741	0.241	0.598	0.607	0.561	0.612	*p* < 0.001 for all

### Motives for risk taking

For the MRT scale, the KMO measure of sampling adequacy was 0.917, Bartlett's test of sphericity was *p* < 0.001 and 61.81% of variance were explained by the items. Twenty-six items were loaded on six factors as follows: the first factor includes motives of irresponsibility (not thinking about the consequences and recklessness); the second factor includes motives of novelty and sensation seeking and the third factor includes hedonistic motives (living in the moment). The fourth factor includes motives of social desirability (getting the attention and care of others, impressing others, etc.), the fifth factor includes achieving future goals and success and the sixth factor includes popularity seeking. Reliability α was 0.909 for the full scale, ranging from 0.874 for searching novelty by risky behaviours to 0.509 for seeking popularity (Table 3).

**Table 3:** MRT items (*n* = 26 items).

Items considered motives for risky behaviours	Factor 1	Factor 2	Factor 3	Factor 4	Factor 5	Factor 6	Cronbach's α
When taking risks, nothing can go wrong	0.794						0.837
I do not care if I have to regret it later	0.775						
Most of the times I do not believe I will get hurt	0.770						
I believe risky behaviours will not hurt me at all	0.760						
I care less about consequences of my behaviours	0.699						
I hate being careful	0.599						
I cannot stay away from these behaviours	0.522						
These behaviours make my heart beat faster		0.974					0.874
These behaviours are thrilling		0.948					
To feel the excitement is wonderful		0.914					
These behaviours give me a kick		0.615					
I do not want to miss enjoying the experience		0.496					
These behaviours give me courage to try new things		0.473					
Trying new things make me happy		0.423					
I love to live the moment			0.887				0.703
Even though I would have to pay, it is important for me to live the moment			0.830				
It is important to enjoy the moment			0.717				
I only live once, and I want to try everything			0.430				
With these behaviours, I get the attention of others				0.940			0.787
These behaviours let other people care for me				0.916			
These behaviours let me impress others				0.734			
Others expect me to behave like this				0.420			
I want to achieve goals that lead to future success					0.930		0.674
I think it is important to achieve goals in the future					0.839		
It is important for me to be popular						0.666	0.509
I enjoy acting ‘cool’						0.541	
Correlation with total scale	0.767	0.885	0.676	0.667	0.279	0.510	*p* < 0.001 for all

### Sample description

In Table 4, we describe the risk perception, attractiveness and motives among boys and girls of Lebanese universities. Boys have more motives and attractiveness for risky behaviours, while girls have a higher perception of danger (*p* < 0.001 for all scales and subscales). There is one exception that is worth noting: girls have higher motives for goals' achievement (*p* = 0.010). This is why in multivariate analysis we kept the RIPS and ARB scales as is, while motives for risky behaviours were analysed separately.

**Table 4:** Risk perception, attractiveness and motives among university students.

Scales and factors	Boys, *n* = 1399 (41.3%)	Girls, *n* = 1980 (58.5%)	*p*-Value	Total, *n* = 3384 (100%)
Has motives for risky behaviours (MRT)	48.10 (17.54)	40.55 (15.63)	< 0.001	43.59 (16.84)
Risk perception (RIPS)	72.49 (19.83)	83.91 (15.57)	< 0.001	79.24 (18.33)
ARB	57.79 (22.44)	47.77 (25.83)	< 0.001	51.96 (24.96)
Irresponsibility (Motive 1)	8.52 (5.68)	6.54 (5.20)	< 0.001	7.34 (5.49)
Novelty seeking (Motive 2)	15.35 (6.60)	11.64 (6.24)	< 0.001	13.14 (6.64)
Seizing the moment (Motive 3)	9.84 (3.81)	9.24 (3.64)	< 0.001	9.48 (3.72)
Social desirability (Motive 4)	4.07 (3.30)	3.00 (2.86)	< 0.001	3.43 (3.09)
Goals achievement (Motive 5)	6.17 (2.09)	6.37 (1.89)	0.010	6.29 (1.98)
Popularity seeking (Motive 6)	3.87 (2.03)	3.61 (2.00)	< 0.001	3.72 (2.02)

### Multivariate analyses for selected risky behaviours

In Table 5, we show the multivariate analyses for selected risky behaviours. A higher RIPS was associated with lower cigarette smoking, cigarette dependence, lower waterpipe dependence and lower problematic alcohol drinking. ARB was associated with higher cigarette smoking, waterpipe smoking and problematic alcohol drinking.

**Table 5:** Multivariate analyses of risky behaviours.

Dependent	Independent variables	Adjusted OR [95% CI]	*p*-Value	Model
Current cigarette smoking	Studying in a private university	1.52 [1.10; 2.10]	0.011	Nagelkerke *R*^2^ = 0.376
	Field of studies (health vs. others)	0.29 [0.18; 0.47]	< 0.001	
	Older age in years	1.08 [1.01; 1.15]	0.036	
	South Lebanon versus others	0.34 [0.17; 0.68]	0.002	
	Mental distress (BDS22)	10.01 [1.00; 1.02]	0.010	
	Female sex versus male	0.26 [0.19; 0.36]	< 0.001	
	Higher socio-economic level	1.22 [1.07; 1.39]	0.003	
	ARB	1.01 [1.00; 1.02]	< 0.001	
	Risk perception (RIPS)	0.99 [0.98; 0.996]	0.003	
	Irresponsibility (Motive 1)	1.08 [1.05; 1.10]	< 0.001	
	Seizing the moment (Motive 3)	1.11 [1.06; 1.16]	< 0.001	
	Goals achievement (Motive 5)	0.93 [0.87; 1.00]	0.052	
Current waterpipe smoking	Field of studies (health vs. others)	0.59 [0.41; 0.85]	0.004	Nagelkerke *R*^2^ = 0.126
	Female sex versus male	0.78 [0.60; 0.96]	0.023	
	Northern Lebanon versus others	0.53 [0.34; 0.83]	0.005	
	ARB	1.01 [1.005; 1.014]	< 0.001	
	Seizing the moment (Motive 3)	1.11 [1.07; 1.15]	< 0.001	
	Social desirability (Motive 4)	1.06 [1.02; 1.10]	0.001	
	Goals achievement (Motive 5)	1.06 [1.00; 1.13]	0.072	
		Adjusted β [95%CI]		
Current cigarette dependence (YACD)	Risk perception (RIPS)	− 0.07 [ − 0.10; − 0.04]	< 0.001	Adjusted *R*^2^ = 0.241
	Irresponsibility (Motive 1)	0.16 [0.05; 0.26]	0.003	
	Social desirability (Motive 4)	0.21 [0.02; 0.40]	0.027	
	Goals achievement (Motive 5)	− 0.38 [ − 0.67; − 0.10]	0.009	
	Popularity seeking (Motive 6)	0.43 [0.17; 0.70]	0.001	
Current waterpipe dependence (LWDS11)	Older age in years	0.65 [0.35; 0.94]	< 0.001	Adjusted *R*^2^ = 0.189
	Female sex versus male	1.36 [0.26; 2.46]	0.016	
	Risk perception (RIPS)	− 0.06 [ − 0.09; − 0.03]	< 0.001	
	Novelty seeking (Motive 2)	0.30 [0.27; 0.38]	< 0.001	
	Goals achievement (Motive 5)	− 0.32 [ − 0.61; − 0.03]	0.031	
Problematic use of alcohol (ASSIST)	Female sex versus male	− 3.50 [ − 4.11; − 2.88]	< 0.001	Adjusted *R*^2^ = 0.260
	Older age in years	− 0.26 [ − 0.40; − 0.11]	< 0.001	
	Socio-economic status	0.81 [0.56; 1.07]	< 0.001	
	Studying in a private university	1.72 [1.13; 2.32]	< 0.001	
	ARB	0.02 [0.01; 0.03]	0.007	
	Risk perception (RIPS)	− 0.02 [ − 0.04; − 0.01]	0.006	
	Irresponsibility (Motive 1)	0.07 [0.003; 0.13]	0.041	
	Novelty seeking (Motive 2)	0.08 [0.01; 0.14]	0.019	
	Seizing the moment (Motive 3)	0.23 [0.13; 0.32]	< 0.001	
	Social desirability (Motive 4)	0.19 [0.09; 0.30]	< 0.001	
	Goals achievement (Motive 5)	− 0.15 [ − 0.30; − 0.04]	0.044	
	Popularity seeking (Motive 6)	− 0.19 [ − 0.33; − 0.04]	0.014	
	Mental distress (BDS22)	0.03 [0.01; 0.05]	0.015	

As for motives for risky behaviours, irresponsibility was associated with more cigarette smoking and dependence and problematic alcohol drinking. Novelty seeking was associated with waterpipe dependence and problematic use of alcohol, while seizing the moment was associated with cigarette smoking, waterpipe smoking and problematic alcohol drinking. Social desirability was associated with waterpipe smoking, cigarette dependence and problematic alcohol drinking. Goals achievement was inversely related to all risky behaviours, while popularity seeking was correlated to cigarette dependence and inversely correlated with problematic alcohol drinking (Table 5).

## Discussion

In this study, we were able to verify the validity and reliability of three scales among Lebanese university students: RIPS, ARB and MRT. Their structures and psychometric properties were not different from the ones found in initial development articles (Kloep et al., 2009; Mantzouranis & Zimmermann, 2010; Shapiro et al., 1998; Siegel et al., 1994).

Moreover, we found that a higher RIPS was associated with lower cigarette smoking, cigarette dependence, lower waterpipe dependence and lower problematic alcohol drinking; risk perception was particularly more common among females. Thus, perceiving the danger of risky behaviours is a driver towards cautiousness among young Lebanese people, similar what is reported by Arab (Al-Kaabba, Saeed, Abdalla, Hassan, & Mustafa, 2011; Shafig et al., 2006) and Chinese researchers (Chueh, Ding, Yao, Huang, & Hung, 2013). These results go in favour of a rational decision-making theory of risk in our population; thus, health education programmes to increase awareness among university students are important to decrease health risk taking behaviours in Lebanon, females in particular. However, our results contradict those of Siegel et al. (1994) and Mantzouranis and Zimmermann (2010), who found that risk perception was not associated with less risky behaviours; moreover, health literacy was not found to be sufficient to decrease risky behaviour (Dermota et al., 2013). This discrepancy may be due to a cultural issue that needs to be addressed in specific studies.

In contrast, ARB was associated with higher cigarette smoking, waterpipe smoking and problematic alcohol drinking: thinking that risky behaviours were attractive was a driver towards adopting these behaviours. These results parallel those of Siegel et al. (1994) and Mantzouranis and Zimmermann (2010), where risky behaviour-associated benefits drive risk intake. However, ARB does not seem to interfere with dependence to cigarettes and waterpipe, which is a concept that includes nicotine addiction and other dimensions that lie beyond individual decision making in young people (Salameh et al., 2008, 2013).

As for motives for risky behaviours, irresponsibility and not thinking about consequences were associated with more cigarette smoking and dependence and problematic alcohol drinking for some individuals; this type of motive was particularly prevalent among males. This goes in line with others' findings (Ben-Zur & Reshef-Kfir, 2003), where risk taking was associated with its perceived benefit without taking into account the negative consequences. Similarly, novelty and sensation seeking was associated with waterpipe dependence and problematic use of alcohol, while seizing the moment was associated with cigarette smoking, waterpipe smoking and problematic alcohol drinking. These types of hedonistic motives have been shown to be associated with cigarette and waterpipe smoking (Hampson, Tildesley, Andrews, Barckley, & Peterson, 2013), alcohol consumption (Weiland et al., 2013), but also with more dangerous behaviours, such as substance misuse (Chakroun, Doron, & Swendsen, 2004) and delinquency (Peach & Gaultney, 2013). Our population, males in particular, could be at risk for this type of behaviour; preventive measures should be taken to avoid these dangerous consequences.

For some individuals, social desirability was associated with waterpipe smoking, cigarette dependence and problematic alcohol drinking, while popularity seeking was correlated to cigarette dependence and inversely correlated with problematic alcohol drinking. The fact that risk taking is associated with making friends and sociability has been shown by several researchers (Amin, Amr, Zaza, & Kaliyadan, 2012; Bonino, Cattelino, & Ciairano, 2005; Dworkin, 2005; Engels & ter Bogt, 2001). Popularity seeking was also shown by Shapiro et al. (1998), in addition to relieving stress perspectives; in fact, we found that mental distress was correlated with higher probability of cigarette smoking and problematic use of alcohol. This association has also been found by Curry and Youngblade (2006), where anger and negative affect directly predicted risky behaviours; other researchers also found similar results (Tavolacci et al., 2013). The role of mental distress remains to be more thoroughly studied in specific studies, perhaps in clinical settings.

In contrast to all risky behaviour motives, goals achievement was significantly more common among females and was inversely related to all assessed risky behaviours. This also goes in favour of a cognitive decision-making theory of risk taking in our population, females in particular. Several studies carried out in the Western world did not find such results: females are currently tending to adopt health-related behaviours similar to males in these societies (Bucksch, Finne, Glücks, Kolip, & HBSC-Team Deutschland, 2012; MacArthur et al., 2012), or even worse (Pritchard & Cox, 2007). In our population, this seems to be another culturally sensitive issue, affected by religiosity and social norms acceptability that still differ between males and females in Arab countries (Sika, 2011).

There is one particular finding in our study that deserves our attention: waterpipe smoking was neither associated with risk perception nor with irresponsibility motive. This corroborates the fact that waterpipe is misconceived as less harmful than cigarettes (Akl et al., 2013; Heinz et al., 2013), and it is frequently consumed among young people (Waked, Salameh, & Aoun, 2009) and women (Salameh et al., 2012b). Similar results were found by Primack et al. (2008). Waterpipe smoking seems to be positioned by university students out of the commonly known risky behaviours. Because waterpipe smoking and dependence have been shown to have negative health consequences (Akl et al., 2010; Salameh et al., 2012c), additional efforts should be made by concerned authorities to increase awareness on health-associated consequences and dangers of waterpipe smoking and dependence.

Our study, as with any, has its limitations: a selection bias could have been possible because the sample is not a random sample and may not be representative of the young adults and students' population in Lebanon. This non-random sampling could lead to an overrepresentation of students who skip classes and may have higher risky behaviours; however, on the other hand, the length of the questionnaire may also lead to an underrepresentation of this students' category, leading to compensation of the latter phenomenon. There could also be a possibility of respondent and information bias because the results of our study are based on a self-administered and completed questionnaire. The cross-sectional nature of the study also precludes causality assessment due to temporality issues.

Despite the fact that we ensured anonymity and confidentiality of all data that have been collected, respondents may have underreported some of their behaviours that lead to missing values. Thus, the assessment of health risk behaviours may have been underestimated in our study. We suggest that further research be carried taking into account these limitations; we also suggest qualitative research that can explore the knowledge, attitudes and values behind the risky behaviours in order to identify their personal, environmental, social and psychological predictors.

## Conclusion

University students in Lebanon, women in particular, demonstrated better perception of risks and wiser associated behaviour than males; all may benefit from heath education programmes to increase their awareness about risky behaviours. A focus on dangers of waterpipe smoking is also suggested because knowledge about its dangers seems deficient. Identifying additional personal, environmental, social and psychological predictors of risky behaviours may also be important to improve effectiveness of these programmes.
